# The impact of stage of labor on adverse maternal and neonatal outcomes in multiparous women: a retrospective cohort study

**DOI:** 10.1186/s12884-020-03286-z

**Published:** 2020-10-07

**Authors:** Li Wang, Hongxia Wang, Lu Jia, Wenjie Qing, Fan Li, Jie Zhou

**Affiliations:** 1 Department of Obstetrics and Gynecology, Hebei General Hospital, Hebei Medical University, Shijiazhuang, 050051 China; 2 Department of Anesthesiology, Perioperative and Pain Medicine, Brigham and Women’s Hospital, Harvard Medical School, 75 Francis St, Boston, MA 02115 USA; 3 Department of Obstetrics and Gynecology, Hebei General Hospital, North China University of Science and Technology, Shijiazhuang, 050051 China; 4 Department of Obstetrics, Chengdu Women’s and Children’s Central Hospital, Chengdu, 610073 China; 5 Department of Otolaryngology-Head and Neck Surgery, Chongqing general hospital, University of Chinese Academy of Science, Chongqing, 40001 China

**Keywords:** First stage of labor, Second stage of labor, Maternal outcomes, Neonatal outcomes, Adverse outcomes, Multiparous women

## Abstract

**Background:**

The correlation between stage of labor and adverse delivery outcomes has been widely studied. Most of studies focused on nulliparous women, it was not very clear what impact the stage of labor duration had on multiparous women.

**Methods:**

A retrospective cohort study was conducted among all the multiparous women of cephalic, term, singleton births, who planned vaginal delivery. The total stage of labor covered the first stage and the second stage in this study, and they were divided into subgroups. Adverse maternal outcomes were defined as referral cesarean delivery, instrumental delivery, postpartum hemorrhage, perineal laceration (III and IV degree), hospitalization stay ≥90th, and adverse neonatal outcomes as NICU, shoulder dystocia, Apgar score ≤ 7(5 min), neonatal resuscitation, assisted ventilation required immediately after delivery.

**Results:**

There were 7109 parturients included in this study. The duration of first stage was 6.2(3.6–10.0) hours, the second stage was 0.3(0.2–0.7) hour, the total stage was 6.9(4.1–10.7) hours in multiparous women. At the first stage, the rates of overall adverse outcome were 21, 23.4, 28.8, 35.5, 38.4% in subgroups < 6 h, 6–11.9 h, 12–17.9 h, 18–23.9 h, ≥24 h, which increased significantly (*X*^2^ = 57.64, *P* < 0.001), and ARR (95% CI) were 1.10 (0.92,1.31), 1.33 (1.04,1.70), 1.80 (1.21,2.68), 2.57 (1.60,4.15) compared with subgroup < 6 h (ARR = 1); At the second stage, the rates of overall adverse outcome were 20.0, 30.7, 38.5, 61.2, 69.6% in subgroups < 1 h, 1–1.9 h, 2–2.9 h, 3–3.9 h, ≥4 h (*X*^*2*^ *=* 349.70*, P* < 0.001), and ARR (95% CI) were 1.89 (1.50, 2.39), 2.22 (1.55, 3.18), 10.64 (6.09, 18.59), 11.75 (6.55, 21.08) compared with subgroup < 1 h (ARR = 1)). At the total stage, the rates of overall adverse outcome were 21.5, 30.8, 42.4% in subgroups < 12 h, 12–23.9 h, ≥24 h (*X*^*2*^ *=* 84.90*, P* < 0.001), and ARR (95% CI) were 1.41 (1.16,1.72), 3.17 (2.10,4.80) compared with subgroup < 12 h (ARR = 1).

**Conclusions:**

The prolonged stage of labor may lead to increased adverse outcomes in multiparous women, it was an independent risk factor of adverse maternal and neonatal outcomes.

## Background

In 2014, in order to reduce the rate of cesarean delivery, the guideline of Safe Prevention of the Primary Cesarean Delivery was recommended by American College of Obstetricians and Gynecologists (ACOG) [1]. The guideline indicated that the prolonged stage of labor may reduce the rate of primary cesarean delivery, and no absolute maximum duration of first and second stage of labor was defined, which was different from Friedman’s Chart [2]. After that, the correlation between stage of labor and adverse delivery outcomes has been widely studied. Most of studies focused on nulliparous women. Some researches supported the original contention that the prolonged second stage beyond historical precepts was unsafe [3, 4]; The implementation of the guideline could not reduce the rate of cesarean delivery, but increased the adverse outcomes of mothers and neonates on the contrary [5], or reduced the rate of primary cesarean delivery successfully, but increased other immediate maternal and neonatal complications [6]; The prolonged first or second stage of labor was related to adverse outcomes [6–9].

It was known that the stage of labor of multiparous women was shorter than that of nulliparous women [10], but it was not very clear what impact the duration of first stage and second stage of labor had on delivery outcomes in multiparous women. We assumed that the prolonged stage of labor might also lead to increased adverse outcomes. In this study, we aimed to evaluate the impact of the duration of stage of labor on delivery outcomes in multiparous women, to further understand the process of labor.

## Methods

This is a retrospective cohort study, the data was collected from the electronic medical records from January 1, 2016 to December 31, 2018 in Harvard University Partners Healthcare Systems (PARTNERS), covering seven hospitals: Brigham and Women’s Hospital, Massachusetts General Hospital, Newton-Wellesley Hospital, North Shore Medical Center, Martha’s Vineyard Hospital, Cooly Dickinson Hospital and Nantucket Cottage Hospital. The collected information of demographics and obstetrics characteristics included gestational age, maternal age, maternal height, weight gain, BMI, gravidity, parity, baby weight, baby height, ethnicity, epidural analgesia, induction, oxytocin, etc., the adverse maternal outcomes included referral cesarean delivery, instrumental delivery, postpartum hemorrhage, III and IV degree laceration, length of stay ≥90th (hospitalization stay ≥90th), and the adverse neonatal outcomes included NICU, shoulder dystocia, Apgar score ≤ 7 (5 min), neonatal resuscitation, assisted ventilation required immediately after delivery.

**Fig. 1:**
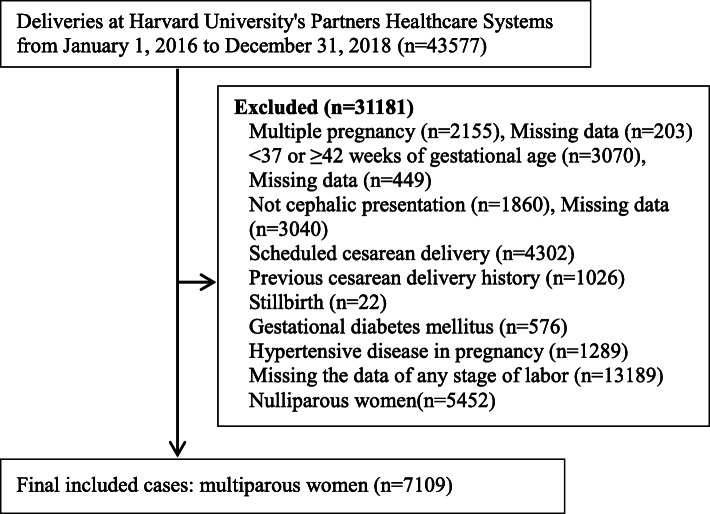
The flow chart

The inclusion criteria was multiparous women (parity ≥1), with singleton gestation, 37–41^+ 6^ gestational weeks, cephalic presentation (excluding face and brow), vaginal delivery, unexpected cesarean delivery during the first or second stage of labor. The exclusion criteria was scheduled cesarean delivery, previous cesarean delivery history, stillbirth, pregnancy induced hypertension, gestational diabetes, and missing data of the duration of first or second stage of labor, or other data. A flow chart was showed in Fig. 1. The first stage of labor was defined as the phase from regular uterine contractions to full cervical dilation. If the cervix had been dilated at the time of admission, the admission time was the beginning of first stage. The second stage was defined as the phase from full cervical dilation to delivery of fetus. The duration of first stage of labor was divided into five subgroups as < 6 h, 6–11.9 h, 12–17.9 h, 18–23.9 h, ≥24 h. The duration of second stage of labor was divided into five subgroups as < 1 h, 1–1.9 h, 2–2.9 h, 3–3.9 h, ≥4 h. The total stage of labor covered the first stage and the second stage, it was divided into three subgroups as < 12 h, 12–23.9 h, ≥24 h.

Data description was presented as mean ± standard deviation (Mean ± SD) or median (interquartile ranges, IQR) for continuous variables and percentages for categorical variables. Linear trend *X*^*2*^ analysis was used for categorical variables as group comparison. The duration of stage of labor were defined as independent variables. The adverse outcomes were defined as dependent variables. Multivariable logistic regression model was used to assess the correlation between the duration of stage of labor and adverse delivery outcomes. Adjusted Risk Ratio (ARR) and 95% Confidence Interval (CI) were used to expressed the association in multivariable logistic models with the shortest duration of the stage of labor (< 6 h of the first stage; < 1 h of the second stage; < 12 h of the total stage) as reference, after adjusting the factors such as gestational age, maternal age, maternal height, weight gain, BMI, gravidity, parity, baby weight, epidural analgesia, induction, oxytocin. All statistical tests of hypotheses will be two sided and criterion for statistical significance is α = 0.05. Statistical analyses were done with SPSS version 21.0 software (IBM).

## Results

There were 43,577 deliveries in Harvard University Partners Healthcare Systems (PARTNERS) from January 1, 2016 to December 31, 2018, including 29,943 vaginal deliveries and 13,634 cesarean deliveries, the rate of cesarean delivery was 31.3%. There were 7109 multiparous women who planned for vaginal delivery and encountered the labor process, 76.3% with epidural analgesia, 27.8% with oxytocin (Table 1). The duration of first stage was 6.2(3.6–10.0) hours in multiparous women, the second stage was 0.3(0.2–0.7) hours, the total stage was 6.9(4.1–10.7) hours. The rate of overall adverse outcomes was 23.6%, the rate of maternal adverse outcomes was 9.4%, the rate of neonatal adverse outcomes was 17.1%, other adverse outcomes were showed in Table 2.

**Table 1:** Demographics and obstetrics characteristics of multiparous women (*n* = 7109)

	Median/n	IQR/%
Duration of stage of labor		
First stage (hour) ^a^	6.2	3.6–10.0
< 6 h	3398	47.8
6–11.9 h	2512	35.3
12–17.9 h	820	11.5
18–23.9 h	228	3.2
≥ 24 h	151	2.1
Second stage (hour) ^a^	0.3	0.2–0.7
< 1 h	5791	81.5
1–1.9 h	776	10.9
2–2.9 h	278	3.9
3–3.9 h	129	1.8
≥ 4 h	135	1.9
Total stage (hour) ^a^	6.9	4.1–10.7
< 12 h	5717	80.4
12–23.9 h	1208	17.0
≥ 24 h	184	2.6
Gestational age (weeks) ^a^	39.6	39.0–40.3
Maternal age (years) ^a^	33.0	30.0–36.0
Maternal Height (cm) ^a^	162.6	160.0–167.6
Gestational weight gain (kg) ^a^	14.0	10.0–17.0
Maternal BMI (kg/m^2^) ^a^	29.4	26.6–32.6
Gravidity^a^	3.0	2.0–4.0
Parity^a^	2.0	2.0–3.0
Baby weight (g) ^a^	3459	3175–3750
Baby height (cm) ^a^	50.8	48.3–52.1
Ethnicity		
Non-Hispanic White	4671	65.7
Non-Hispanic Black	558	7.9
Hispanic	273	3.8
Asian or Pacific Islanders	737	10.4
Other or Unknown	870	12.2
Epidural analgesia	5423	76.3
Induction	1647	23.2
Oxytocin	1975	27.8

Data was presented as n (%) or ^a^median (interquartile range, non-normal distribution)

**Table 2:** Adverse delivery outcomes in multiparous women

	n	%
Overall outcomes	1677	23.6
Maternal outcomes	671	9.4
Referral cesarean delivery	98	1.4
Instrumental delivery	207	2.9
Postpartum hemorrhage	155	2.2
III and IV degree laceration	78	1.1
Length of stay ≥90th	332	4.7
Neonatal outcomes	1213	17.1
NICU	411	5.8
Shoulder dystocia	185	2.6
Apgar ≤7(5 min)	89	1.3
Neonatal resuscitation	790	11.1
Assisted ventilation	181	2.5

Overall outcomes include maternal and neonatal adverse outcomes

**Fig. 2:**
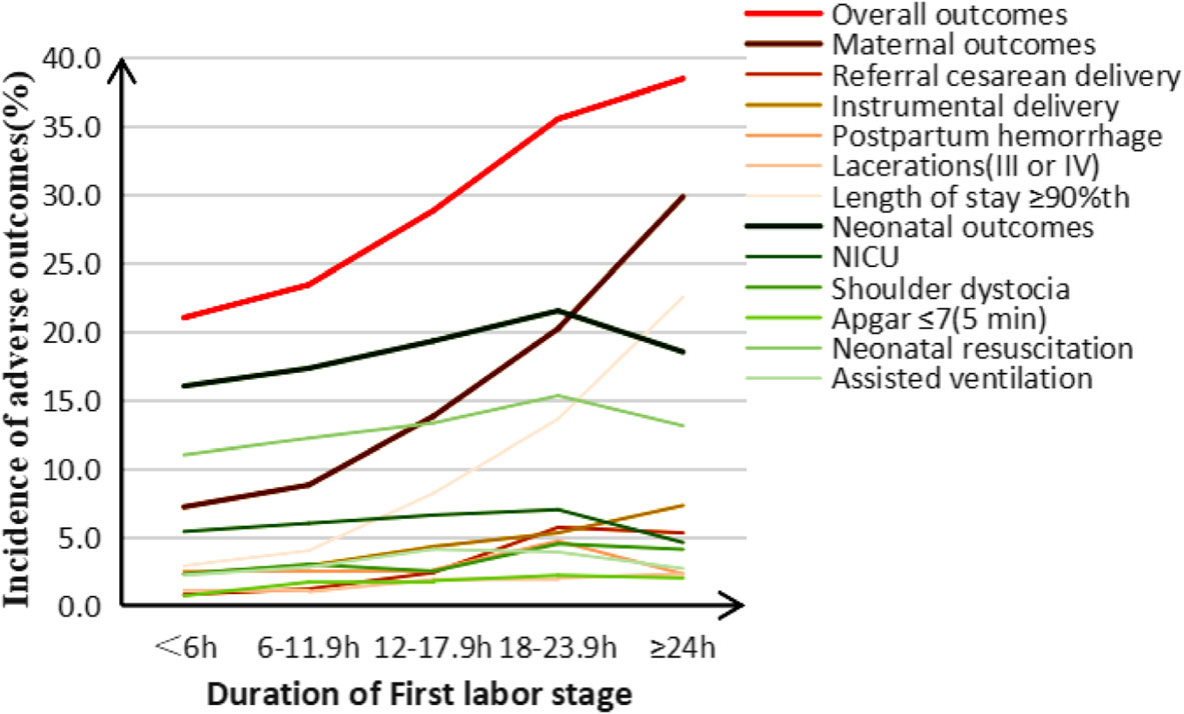
Adverse outcomes in the first stage of labor in multiparous women

**Fig. 3:**
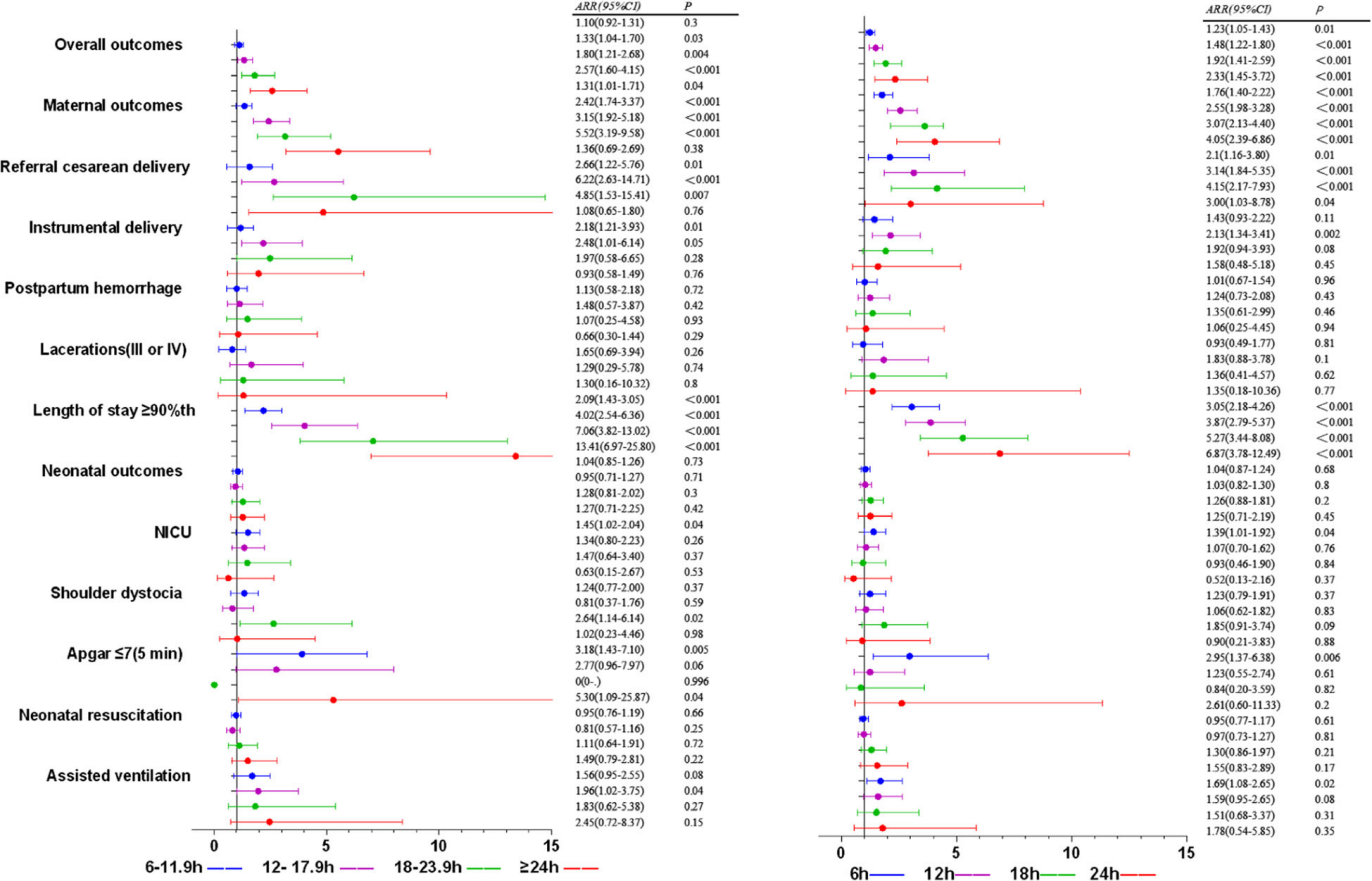
Forest plots for multivariable logistic regression model of the first stage of labor. To assess the relationship between the duration of stage of labor and adverse delivery outcomes, the left was compared with < 1 h, the right was compared with cutoff value. Adjusted gestational age, maternal age, maternal height, maternal BMI, gravidity, parity, baby weight, baby height, epidural, anesthesia, induction, oxytocin

With the prolongation of first stage, the rates of overall adverse outcomes were 21, 23.4, 28.8, 35.5, 38.4% in subgroups < 6 h, 6–11.9 h, 12–17.9 h, 18–23.9 h, ≥24 h, the rates increased significantly (*X*^*2*^ = 57.64, *P* < 0.001). The rate of maternal adverse outcomes was 7.2, 8.8, 13.8, 20.2, 29.8% respectively, which increased significantly (*X*^*2*^ = 121.38, *P* < 0.001); The rate of neonatal adverse outcomes was 16.0, 17.3, 19.3, 21.5, 18.5% respectively, which increased significantly (*X*^*2*^ = 7.75, *P* = 0.005). There were significant differences in the incidence of referral cesarean delivery, instrumental delivery, length of stay ≥90th, shoulder dystocia, Apgar ≤7(5 min), neonatal resuscitation, assisted ventilation in different duration of the first stage (Table 3, Fig. 2). In order to assess the effect of duration of labor on adverse outcomes, we made the following analysis, with < 6 h as the reference. Multivariable logistic regression showed that ARR(95%CI) of overall adverse outcomes were 1.10(0.92,1.31), 1.33(1.04,1.70), 1.80(1.21,2.68), 2.57(1.60,4.15) in subgroups of 6–11.9 h, 12–17.9 h, 18–23.9 h, ≥24 h; ARR(95% CI) of maternal adverse outcomes were 1.31(1.01,1.71), 2.42(1.74,3.37), 3.15(1.92,5.18), 5.52(3.19,9.58); ARR(95% CI) of neonatal adverse outcomes were 1.04(0.85,1.26), 0.95(0.71,1.27), 1.28(0.81,2.02), 1.27(0.71,2.25). The risk of referral cesarean delivery and length of stay ≥90th increased with prolonged first stage. This was showed in Additional file 1: Table S1, Fig. 3. In order to understand the impact of different cutoff values on the risk of overall adverse outcomes, the first stage < 6 h and ≥ 6 h were compared, ARR(95% CI) was 1.23(1.05, 1.43); if the cutoff values were 12 h, 18 h, 24 h, ARR(95% CI) were 1.48(1.22, 1.80), 1.92(1.41, 2.59), 2.33(1.45, 3.72); ARR(95% CI) of maternal adverse outcomes were 1.76(1.40, 2.22), 2.55(1.98, 3.28), 3.61(2.13, 4.40), 4.05(2.39, 6.86), ARR(95% CI) of neonatal adverse outcomes were 1.04(0.87,1.24), 1.03(0.82,1.30), 1.26(0.88,1.81), 1.25(0.71,2.19) with 6 h,12 h, 18 h, 24 h as cutoff values. The risk of referral cesarean delivery and length of stay ≥90th increased with the change of cutoff values. Other outcomes were showed in Additional file 2: Table S2, Fig. 3.

**Table 3:** Adverse outcomes in the first stage of labor in multiparous women (n = 7109)

	Duration of First stage of labor					*Linear trend X*^*2*^	*P*
	< 6 h	6–11.9 h	12–17.9 h	18–23.9 h	≥24 h		
Overall outcomes	714(21.0)	588(23.4)	236(28.8)	81(35.5)	58(38.4)	57.64	< 0.001
Maternal outcomes	246(7.2)	221(8.8)	113(13.8)	46(20.2)	45(29.8)	121.38	< 0.001
Referral cesarean delivery	28(0.8)	29(1.2)	20(2.4)	13(5.7)	8(5.3)	50.97	< 0.001
Instrumental delivery	77(2.3)	72(2.9)	35(4.3)	12(5.3)	11(7.3)	23.46	< 0.001
Postpartum hemorrhage	72(2.5)	53(2.5)	18(2.6)	9(4.7)	3(2.3)	0.73	0.393
III and IV degree laceration	34(1.1)	23(1.0)	14(1.9)	4(2.0)	3(2.3)	3.76	0.053
Length of stay ≥90th	100(2.9)	100(4.0)	67 (8.2)	31(13.6)	34(22.5)	160.81	< 0.001
Neonatal outcomes	543(16.0)	435(17.3)	158(19.3)	49(21.5)	28(18.5)	7.75	0.005
NICU	184(5.4)	150(6.0)	54(6.6)	16(7.0)	7(4.6)	1.14	0.285
Shoulder dystocia	76(2.3)	73(3.0)	20(2.5)	10(4.5)	6(4.1)	4.33	0.037
Apgar ≤7(5 min)	24(0.7)	42(1.7)	15(1.8)	5(2.2)	3(2.0)	12.66	< 0.001
Neonatal resuscitation	354(11.0)	287(12.2)	101(13.3)	31(15.3)	17(13.1)	5.77	0.016
Assisted ventilation	72(2.2)	66(2.8)	31(4.1)	8(3.9)	4(2.7)	7.13	0.008

Data was presented as n(%). Linear trend Chi-square test for categorical data

**Fig. 4:**
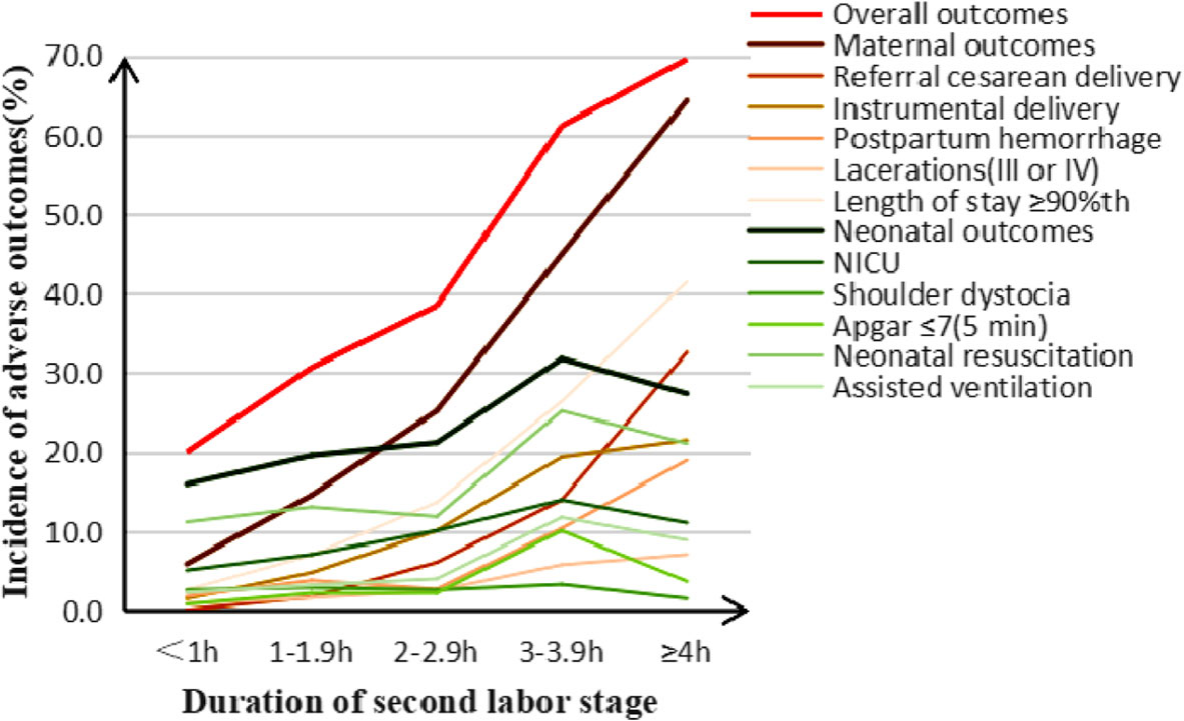
Adverse outcomes in the second stage of labor in multiparous women

**Fig. 5:**
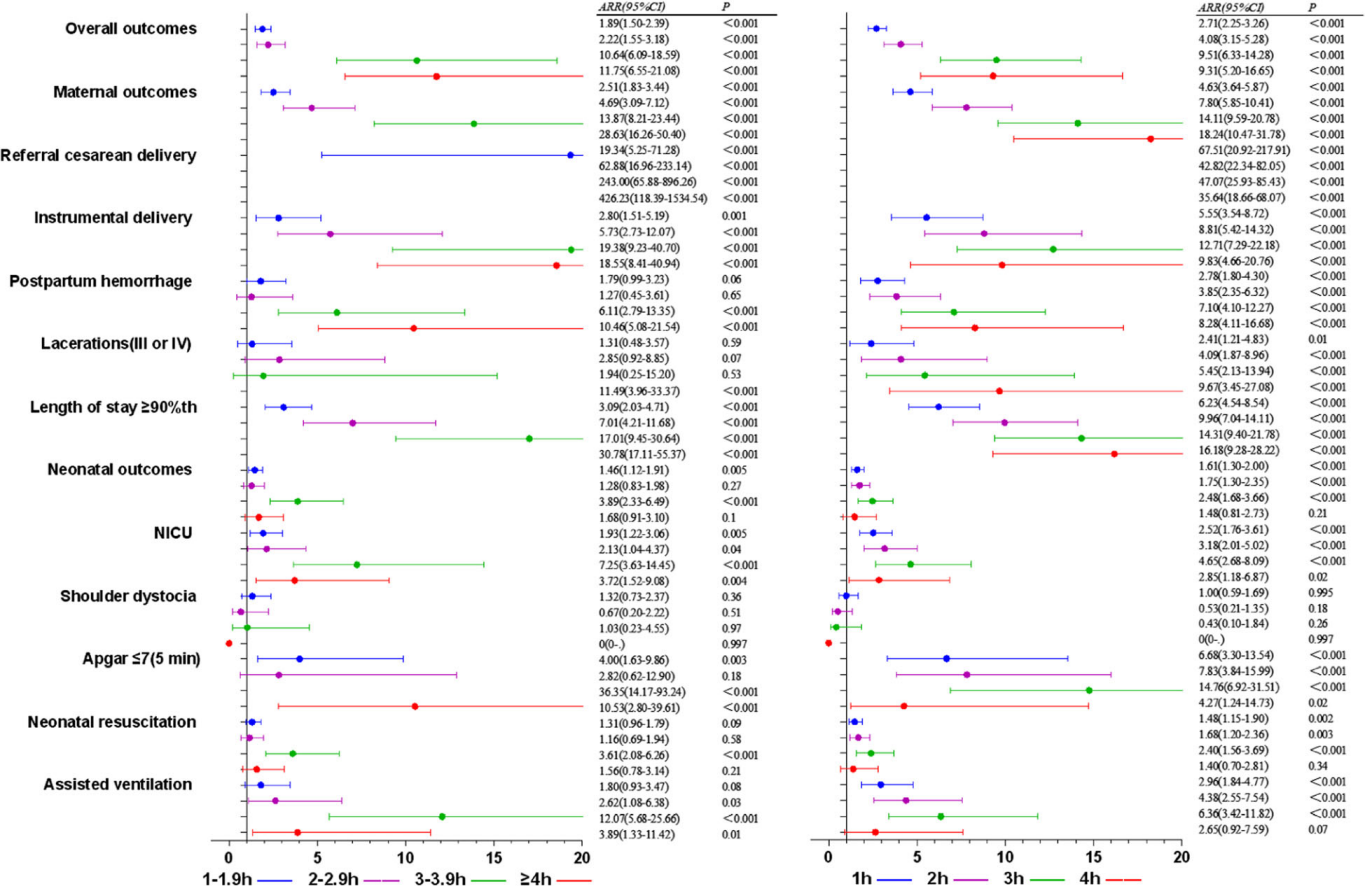
Forest plots for multivariable logistic regression model of the second stage of labor. To assess the relationship between the duration of stage of labor and adverse delivery outcomes, the left was compared with < 1 h, the right was compared with cutoff value. Adjusted gestational age, maternal age, maternal height, maternal BMI, gravidity, parity, baby weight, baby height, epidural, anesthesia, induction, oxytocin

With the prolongation of second stage of labor, the rates of overall adverse outcomes were 20.0, 30.7, 38.5, 61.2, 69.6% in subgroups < 1 h, 1–1.9 h, 2–2.9 h, 3–3.9 h, ≥4 h (*X*^*2*^ *=* 349.70*, P* < 0.001). The rate of maternal adverse outcomes increased from 5.9, 14.6, 25.2, 45.0, to 64.4% (*X*^*2*^ *=* 821.97*, P* < 0.001); The rate of neonatal adverse outcomes was 16.0, 19.6, 21.2, 31.8, 27.4%, which increased significantly (*X*^*2*^ *=* 38.06*, P* < 0.001). There were significant differences in the rate of referral cesarean delivery, instrumental delivery, postpartum hemorrhage, III and IV degree laceration, length of stay ≥90th, NICU, Apgar ≤7(5 min), neonatal resuscitation, assisted ventilation in second stage of labor (Table 4, Fig. 4). With < 1 h as the reference group of second stage, ARR (95% CI) of 1–1.9 h, 2–2.9 h, 3–3.9 h, 4 h were 1.89(1.50, 2.39), 2.22 (1.55, 3.18), 10.64 (6.09, 18.59), 11.75(6.55, 21.08) in overall adverse outcomes; ARR(95%CI) were 2.51(1.83,3.44), 4.69(3.09,7.12), 13.87(8.21,23.44), 28.63(16.26,50.40) in maternal outcomes; ARR(95%CI) were 1.46(1.12,1.91), 1.28(0.83,1.98), 3.89(2.33,6.49), 1.68(0.91,3.10) in neonatal outcomes; other outcomes showed in Additional file 3: Table S3, Fig. 5. Regarding the impact of cutoff values of second stage on the risk of adverse outcomes, ARR (95% CI) of overall adverse outcomes were 2.71(2.25, 3.26), 4.08(3.15, 5.28), 9.51(6.33, 14.28), 9.31(5.20, 16.65) with 1 h, 2 h, 3 h, 4 h as the cutoff values; ARR (95% CI) of maternal adverse outcomes were 4.63(3.64,5.87), 7.80(5.85,10.41), 14.11(9.59,20.78), 18.24(10.47,31.78); ARR (95% CI) of neonatal adverse outcomes were 1.61(1.30,2.00), 1.75(1.30,2.35), 2.48(1.68,3.66), 1.48(0.81,2.73). Other adverse outcomes were showed in Additional file 4: Table S4, Fig. 5.

**Table 4:** Adverse outcomes in the second stage of labor in multiparous women (n = 7109)

	Duration of second stage of labor					*Linear trend X*^*2*^	*P*
	< 1 h	1–1.9 h	2–2.9 h	3–3.9 h	≥4 h		
Overall outcomes	1159(20.0)	238(30.7)	107(38.5)	79(61.2)	94(69.6)	349.70	< 0.001
Maternal outcomes	343(5.9)	113(14.6)	70(25.2)	58(45.0)	87(64.4)	821.97	< 0.001
Referral cesarean delivery	6(0.1)	13(1.7)	17(6.1)	18(14.0)	44(32.6)	989.84	< 0.001
Instrumental delivery	88(1.5)	37(4.8)	28(10.1)	25(19.4)	29(21.5)	375.02	< 0.001
Postpartum hemorrhage	90(1.8)	25(3.8)	6(2.7)	12(10.4)	22(19.0)	130.31	< 0.001
III and IV degree laceration	48(0.9)	11(1.6)	6(2.5)	6(5.8)	7(7.1)	46.88	< 0.001
Length of stay≥90th	150(2.6)	54(7.0)	38(13.7)	34(26.4)	56(41.5)	614.33	< 0.001
Neonatal outcomes	924(16.0)	152(19.6)	59(21.2)	41(31.8)	37(27.4)	38.06	< 0.001
NICU	295(5.1)	55(7.1)	28(10.1)	18(14.0)	15(11.1)	36.42	< 0.001
Shoulder dystocia	150(2.6)	22(2.9)	7(2.6)	4(3.3)	2(1.5)	0.05	0.83
Apgar ≤7(5 min)	48(0.8)	17(2.2)	6(2.2)	13(10.1)	5(3.7)	64.22	< 0.001
Neonatal resuscitation	609(11.2)	95(13.1)	30(11.9)	30(25.2)	26(21.1)	24.22	< 0.001
Assisted ventilation	123(2.3)	23(3.2)	10(4.0)	14(11.8)	11(8.9)	48.11	< 0.001

Data was presented as n(%). Linear trend Chi-square test for categorical data

**Fig. 6:**
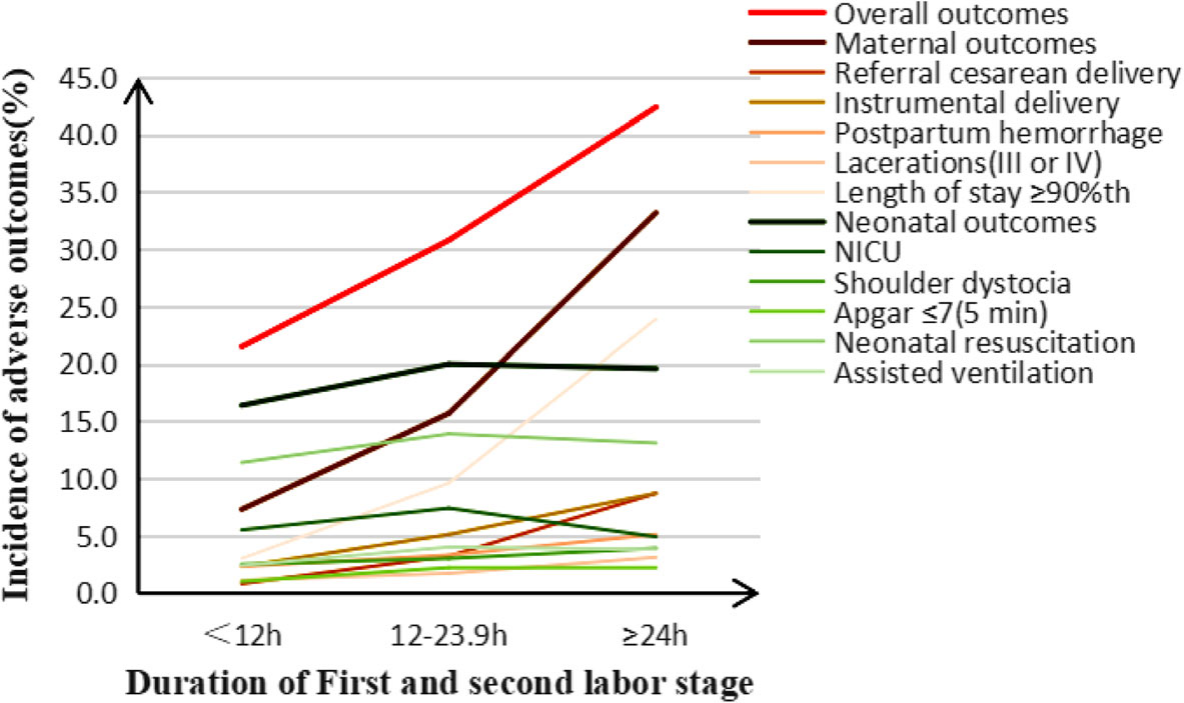
Adverse outcomes in the total stage of labor in multiparous women

**Fig. 7:**
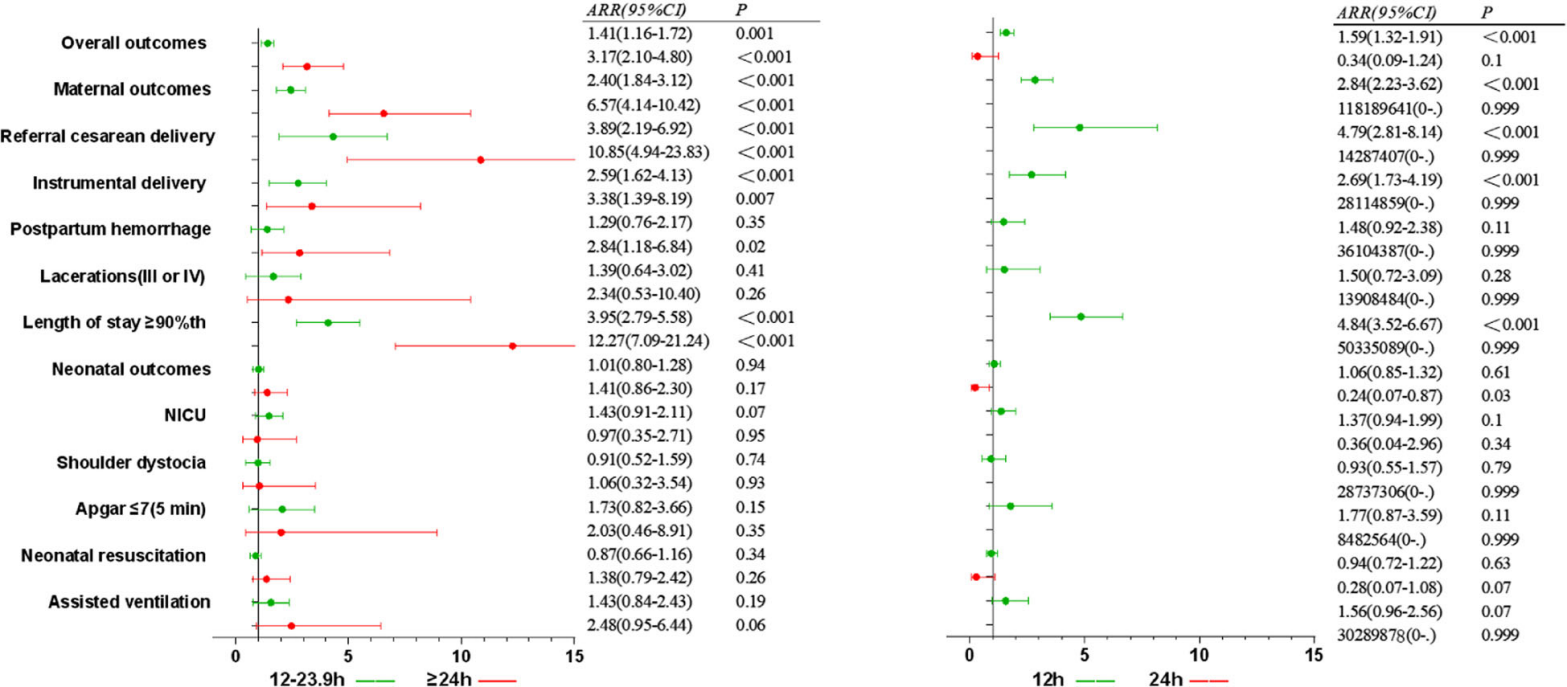
Forest plots for multivariable logistic regression model of the total stage of labor. To assess the relationship between the duration of stage of labor and adverse delivery outcomes, the left was compared with < 12 h, the right was compared with cutoff value. Adjusted gestational age, maternal age, maternal height, maternal BMI, gravidity, parity, baby weight, baby height, epidural, anesthesia, induction, oxytocin

The total stage of labor was stratified to < 12 h, 12–23.9 h, and ≥ 24 h. With the increase of total stage of labor, the rate of overall adverse outcomes were 21.5, 30.8, 42.4% (*X*^*2*^ *=* 84.90*, P* < 0.001); the rate of material adverse outcomes were 7.3, 15.7, 33.2% (*X*^*2*^ = 195.48, *P* < 0.001); the rate of neonatal adverse outcomes were 16.4, 20.0, 19.6% (*X*^*2*^ *=* 8.61, *P* = 0.003). The rate was (significantly) different in terms of referral cesarean delivery, instrumental delivery, postpartum hemorrhage, III and IV degree laceration, length of stay ≥90th, Apgar ≤7(5 min), neonatal resuscitation, and assisted ventilation (Table 5, Fig. 6). With total stage of labor < 12 h as the reference, the risk of overall adverse outcomes, ARR (95% CI) were 1.41(1.16,1.72), 3.17 (2.10,4.80) in subgroups of 12–23.9 h, ≥24 h, the risk of maternal outcomes, ARR (95% CI) was 2.40 (1.84,3.12), 6.57 (4.14,10.42), and the risk of neonatal outcomes, ARR (95% CI) was 1.01 (0.80,1.28), 1.41 (0.86,2.30), (Additional file 5: Table S5, Fig. 7). Regarding the impact of the total stage on the risk of overall adverse outcomes, ARR (95% CI) were 1.59(1.32,1.91) with 12 h, 24 h as the cutoff values; ARR (95% CI) of maternal adverse outcome was 2.84(2.23,3.62) with 12 h as the cutoff value, but there was no effect on the neonatal adverse outcomes (Additional file 6: Table S6, Fig. 7).

**Table 5:** Adverse outcomes in the total stage of labor in multiparous women. (*n* = 7109)

	Duration of total stage of labor			*Linear trend X*^*2*^	*P*
	< 12 h	12–23.9 h	≥24 h		
Overall outcomes	1227(21.5)	372(30.8)	78(42.4)	84.90	< 0.001
Maternal outcomes	420(7.3)	190(15.7)	61(33.2)	195.48	< 0.001
Referral cesarean delivery	43(0.8)	39(3.2)	16(8.7)	111.98	< 0.001
Instrumental delivery	129(2.3)	62(5.1)	16(8.7)	51.34	< 0.001
Postpartum hemorrhage	113(2.3)	34(3.3)	8(5.1)	6.86	0.009
III and IV degree laceration	55(1.1)	18(1.7)	5(3.1)	6.96	0.008
Length of stay≥90th	172(3.0)	116(9.6)	44(23.9)	239.48	< 0.001
Neonatal outcomes	936(16.4)	241(20.0)	36(19.6)	8.61	0.003
NICU	313(5.5)	89(7.4)	9(4.9)	2.91	0.088
Shoulder dystocia	143(2.5)	35(3.0)	7(3.9)	1.68	0.194
Apgar ≤7(5 min)	58(1.0)	27(2.2)	4(2.2)	11.83	0.001
Neonatal resuscitation	615(11.4)	154(13.9)	21(13.1)	4.62	0.032
Assisted ventilation	131(2.4)	44(4.0)	6(3.8)	7.70	0.006

Data was presented as n(%). Linear trend Chi-square test for categorical data

## Discussion

There has been a constant debate on the duration of stage of labor. In decades, Friedman’s Chart has been used to assist in the management of labor [2], and the duration of the stage of labor was defined based on it. Zhang and others proposed a new delivery curve [11], that was referred by the ACOG consensus in 2014. Then Cohen and Friedman claimed that the new curve incorrectly explained the Friedman curve [12]. Studies after 2014 [5, 6] showed that prolonged stage of labor would not reduce the rate of cesarean section delivery, but would increase the adverse outcomes of delivery. Most of these studies tended to classify the stage of labor into normal and abnormal stage, with the intention of implementation of intervention. The stage of labor of multiparous women was different from that of nulliparous women. After the promulgation of the consensus in 2014, we wanted to figure out the impact of stage of labors of multiparous women on the adverse delivery outcomes. There was 5.3% of multiparous women of first stage ≥18 h, 3.7% of second stage ≥3 h, and 2.6% of total stage ≥24 h, these durations were not recommended neither by the ACOG consensus nor by Friedman’s Chart. When the overall rate of cesarean delivery was 31.29%, the rate of referral cesarean deliveries was 1.4%, instrumental vaginal deliveries was 2.9% in this study.

The prolonged first stage of labor increased adverse maternal and neonatal outcomes in multiparous women in this study. With the prolongation of first stage, the rate of overall adverse outcomes increased from 21.0 to 38.4%, both maternal and neonatal adverse outcomes showed an increasing trend. Compared with (the subgroup) < 6 h of first stage, the risks of long time stay in hospital, low Apgar score and admission to NICU increased in the subgroup of 6–11.9 h, the risks of assisted ventilation, referral cesarean delivery and instrumental delivery increased in the subgroup of 12–17.9 h, the risks of long time stay in hospital and referral cesarean delivery increased in the subgroup ≥18 h, even if confounder factors such as birth weight were adjusted. The risks of referral cesarean delivery, hospitalization stay ≥90th were 3.15 times and 4.27 times in subgroup ≥18 h of that in subgroup < 18 h of first stage. Therefore, we should comprehensively evaluate the possibility of vaginal delivery and the adverse outcomes, especially (when the duration of stage of labor is) ≥18 h. Zhang and his colleagues pointed out that “our failure to reduce the rate of cesarean section may be due to our failure to fully understand the delivery process, especially the first stage of labor” [13]. We should take a dynamic view on the first stage, define an abnormal first stage based on maternal and neonatal outcomes [7, 14], and pay attention to complications even at the beginning of labor.

The second stage was the most important stage of labor. The effect of the duration of the second stage of labor on the outcomes of labor has been studied widely and deeply, but the results were not consistent. Grantz’s study showed the rate of spontaneous vaginal birth without morbidity decreased with prolonged duration of second stage [15]. Cheng YW and Laughon SK proposed that the benefits of the prolonged second stage of labor to promote the rate of vaginal delivery should be weighed against the increased adverse outcomes of mothers and infants [16, 17]. A randomized controlled study in 2016 showed that the prolonged second stage of labor could promote the rate of vaginal delivery and reduce the rate of cesarean section, but the impact of the prolonged second stage of labor on the adverse outcomes of mothers and infants was not statistically significant [18]. Thuillier C and Zipori Y reported the new consensus recommendations was associated with the reduction of the rate of primary cesarean delivery [6, 19]. Ausbeck EB’s research showed prolonged second stage was associated with adverse maternal outcomes significantly, but not with adverse neonatal outcomes [20].

In this study, the rate of overall adverse outcomes increased rapidly from 20% in subgroup ≤1 h, 30.7% in 1–1.9 h, 38.5% in 2–2.9 h, to 61.2% in 3–3.9 h, 69.6% in ≥4 h of second stage of labor. Except for shoulder dystocia, almost all adverse outcomes showed an increasing trend. Although the risk of shoulder dystocia increased with birth weight, and was related to prolonged stage of labor [21–23], but in this study, the shoulder dystocia reduced when the duration of second stage ≥4 h, it might be owing to the increase of referral cesarean delivery with prolonged second stage and decrease of vaginal deliveries. The risk of maternal adverse outcomes increased for every additional hour, in referral cesarean delivery, instrumental delivery, postpartum hemorrhage, lacerations, hospitalization stay ≥90th. It also should be noticed that the prolonged second stage was an independent risk factor for adverse neonatal outcomes, in low Apgar score at 5 min, neonatal resuscitation, assisted ventilation and admission to NICU, even though that was not significant in the first stage of labor.

The duration of second stage did not account much in the total stage, but more attention should be pay to it. Some harmless measures should be taken to make it shorter, such as upright position or immediate pushing [24, 25]. It was important to assess the fetal position in second stage, occiput posterior position and transverse position were associated with more pain and prolonged stage of labor. Free position or manual rotation of fetal occiput in setting of fetal malposition in second stage of labor were reasonable interventions before instrument-assisted delivery or cesarean delivery [1, 26, 27].

Few studies have paid attention to total stage of labor. In this study, the rate of overall adverse outcomes was 21.5, 30.8, 42.4%, in subgroups < 12 h, 12–17.9 h, ≥18 h. The risk of postpartum hemorrhage with the duration of total ≥ 24 h was 1.84 times of that with the duration < 12 h. There were correlation between the first and second stages of labor, the duration of the second stage significantly increased accordingly with the increase of duration of the first stage [28]. In the first stage, the risk factors should be identified and treated, such as uterine atony, fatigue, insufficient energy intake, supine position and so on. With the duration of first stage ≥18 h, we should comprehensively weigh the possibility of vaginal delivery against the incidence of adverse outcomes. Especially with cephalopelvic disproportion or fetal distress, it should be referred to cesarean section in time. When the first stage was prolonged, we must be alert to the prolongation of the second stage, and pay more attention to the prevention of postpartum complications in parturients.

The strength of the study was that the adverse maternal and neonatal outcomes increased with prolongation of the first, second and total stage of labor, the longer the duration of stage, the higher the risk of adverse outcomes in multiparous women. The limitation of the study was that the data was collected retrospectively; the first stage of labor could not be classified into latent phase and active phase. The time points could not be defined very clearly from the first stage to the second sage, because the examination of dilated cervix was subjective. There was not a stratified analysis on epidural analgesia, induction and oxytocin.

## Conclusion

The prolonged stage of labor may lead to increased adverse outcomes in multiparous women, no matter the first stage, the second stage or the total stage of labor, and it was an independent risk factor for adverse maternal and neonatal outcomes, increasing synchronously. The impact of prolonged stage of labor on maternal outcomes was more significant, and the prolonged second stage had an impact on neonatal outcomes. We suggest to pay attention to the stage of labor at the beginning, monitor the status of mothers and neonates, and take active measures to make stage of labor shorter and thus to reduce adverse delivery outcomes.

## Supplementary information

**Additional file 1: Table S1** Risks of adverse outcomes in the first stage of labor in multiparous women. Multivariable logistic regression model was used to assess the relationship between the stage of labor and adverse delivery outcomes, < 6 h as a reference; Adjusted gestational age, maternal age, maternal height, maternal BMI, gravidity, parity, baby weight, baby height, epidural, anesthesia, induction, oxytocin.

**Additional file 2: Table S2** Risks of adverse outcomes in different cutoff value of the first stage of labor in multiparous women. Multivariable logistic regression model was used to assess risks of cutoff value of the stage of labor for adverse delivery outcomes. Adjusted gestational age, maternal age, maternal height, maternal BMI, gravidity, parity, baby weight, baby height, epidural, anesthesia, induction, oxytocin.

**Additional file 3: Table S3** Risks of adverse outcomes in the second stage of labor in multiparous women. Multivariable logistic regression model was used to assess the relationship between the stage of labor and adverse delivery outcomes, < 1 h as a reference; Adjusted gestational age, maternal age, maternal height, maternal BMI, gravidity, parity, baby weight, baby height, epidural, anesthesia, induction, oxytocin.

**Additional file 4: Table S4** Risks of adverse outcomes in different cutoff value of the second labor stage in multiparous women. Multivariable logistic regression model was used to assess risks of cutoff value of the stage of labor for adverse delivery outcomes. Adjusted gestational age, maternal age, maternal height, maternal BMI, gravidity, parity, baby weight, baby height, epidural, anesthesia, induction, oxytocin.

**Additional file 5: Table S5** Risks of adverse outcomes in the total stage of labor in multiparous women. Multivariable logistic regression model was used to assess the relationship between the stage of labor and adverse delivery outcomes, < 12 h as a reference; Adjusted gestational age, maternal age, maternal height, maternal BMI, gravidity, parity, baby weight, baby height, epidural, anesthesia, induction, oxytocin.

**Additional file 6: Table S6** Risks of adverse outcomes in different cutoff value of the total stage of labor in multiparous women. Multivariable logistic regression model was used to assess risks of cutoff value of the stage of labor for adverse delivery outcomes. Adjusted gestational age, maternal age, maternal height, maternal BMI, gravidity, parity, baby weight, baby height, epidural, anesthesia, induction, oxytocin.

## Data Availability

The datasets used in the current study are available from the corresponding author on reasonable request.

## Abbreviations

ARR: Adjusted Risk Ratio
CI: Confidence Interval
ACOG: American College of Obstetricians and Gynecologists
