# Supplementary material for: The impact of stage of labor on adverse maternal and neonatal outcomes in multiparous women: a retrospective cohort study
Source: BMC Pregnancy Childbirth. 2020 Oct 7;20:596. doi: 10.1186/s12884-020-03286-z (PMC7542423; doi:10.1186/s12884-020-03286-z)
Supplement: Supplementary file 3 — Additional file 3: Table S3 Risks of adverse outcomes in the second stage of labor in multiparous women. Multivariable logistic regression model was used to assess the relationship between the stage of labor and adverse delivery outcomes, < 1 h as a reference; Adjusted gestational age, maternal age, maternal height, maternal BMI, gravidity, parity, baby weight, baby height, epidural, anesthesia, induction, oxytocin. [file 12884_2020_3286_MOESM3_ESM.doc]

Table S3 Risks of adverse outcomes in the second stage of labor in multiparous women.

| Duration of the second stage of labor n(%) | | | | | | | | |
| --- | --- | --- | --- | --- | --- | --- | --- | --- |
|  | 1-1.9h |  | 2-2.9h |  | 3-3.9h |  | ≥4h |  |
|  | ARR(95%CI) | P | ARR(95%CI) | P | ARR(95%CI) | P | ARR(95%CI) | P |
| Overall outcomes | 1.89(1.50,2.39) | <0.001 | 2.22(1.55,3.18) | <0.001 | 10.64(6.09,18.59) | <0.001 | 11.75(6.55,21.08) | <0.001 |
| Maternal outcomes | 2.51(1.83,3.44) | <0.001 | 4.69(3.09,7.12) | <0.001 | 13.87(8.21,23.44) | <0.001 | 28.63(16.26,50.40) | <0.001 |
| Referral cesarean delivery | 19.34(5.25,71.28) | <0.001 | 62.88(16.96,233.14) | <0.001 | 243.00(65.88,896.26) | <0.001 | 426.23(118.39,1534.54) | <0.001 |
| Instrumental delivery | 2.80(1.51,5.19) | 0.001 | 5.73(2.73,12.07) | <0.001 | 19.38(9.23,40.70) | <0.001 | 18.55(8.41,40.94) | <0.001 |
| Postpartum hemorrhage | 1.79(0.99,3.23) | 0.06 | 1.27(0.45,3.61) | 0.65 | 6.11(2.79,13.35) | <0.001 | 10.46(5.08,21.54) | <0.001 |
| III and IV degree laceration | 1.31(0.48,3.57) | 0.59 | 2.85(0.92,8.85) | 0.07 | 1.94(0.25,15.20) | 0.53 | 11.49(3.96,33.37) | <0.001 |
| Length of stay ≥90th | 3.09(2.03,4.71) | <0.001 | 7.01(4.21,11.68) | <0.001 | 17.01(9.45,30.64) | <0.001 | 30.78(17.11,55.37) | <0.001 |
| Neonatal outcomes | 1.46(1.12,1.91) | 0.005 | 1.28(0.83,1.98) | 0.27 | 3.89(2.33,6.49) | <0.001 | 1.68(0.91,3.10) | 0.10 |
| NICU | 1.93(1.22,3.06) | 0.005 | 2.13(1.04,4.37) | 0.04 | 7.25(3.63,14.45) | <0.001 | 3.72(1.52,9.08) | 0.004 |
| Shoulder dystocia | 1.32(0.73,2.37) | 0.36 | 0.67(0.20,2.22) | 0.51 | 1.03(0.23,4.55) | 0.97 | 0.00(0.00,.) | 0.997 |
| Apgar ≤7(5 min) | 4.00(1.63,9.86) | 0.003 | 2.82(0.62,12.90) | 0.18 | 36.35(14.17,93.24) | <0.001 | 10.53(2.80,39.61) | <0.001 |
| Neonatal resuscitation | 1.31(0.96,1.79) | 0.09 | 1.16(0.69,1.94) | 0.58 | 3.61(2.08,6.26) | <0.001 | 1.56(0.78,3.14) | 0.21 |
| Assisted ventilation | 1.80(0.93,3.47) | 0.08 | 2.62(1.08,6.38) | 0.03 | 12.07(5.68,25.66) | <0.001 | 3.89(1.33,11.42) | 0.01 |

Multivariable logistic regression model was used to assess the relationship between the stage of labor and adverse delivery outcomes, <1h as a reference; Adjusted gestational age, maternal age, maternal height, maternal BMI, gravidity, parity, baby weight, baby height, epidural, anesthesia, induction, oxytocin.
