# Supplementary material for: The impact of stage of labor on adverse maternal and neonatal outcomes in multiparous women: a retrospective cohort study
Source: BMC Pregnancy Childbirth. 2020 Oct 7;20:596. doi: 10.1186/s12884-020-03286-z (PMC7542423; doi:10.1186/s12884-020-03286-z)
Supplement: Supplementary file 4 — Additional file 4: Table S4 Risks of adverse outcomes in different cutoff value of the second labor stage in multiparous women. Multivariable logistic regression model was used to assess risks of cutoff value of the stage of labor for adverse delivery outcomes. Adjusted gestational age, maternal age, maternal height, maternal BMI, gravidity, parity, baby weight, baby height, epidural, anesthesia, induction, oxytocin. [file 12884_2020_3286_MOESM4_ESM.doc]

Table S4 Risks of adverse outcomes in different cutoff value of the second labor stage in multiparous women.

| Duration of the second stage of labor n(%) | | | | | | | | |
| --- | --- | --- | --- | --- | --- | --- | --- | --- |
|  | 1h |  | 2h |  | 3h |  | 4h |  |
|  | *ARR(95%CI)* | *P* | *ARR(95%CI)* | *P* | *ARR(95%CI)* | *P* | *ARR(95%CI)* | *P* |
| Overall outcomes | 2.71(2.25,3.26) | <0.001 | 4.08(3.15,5.28) | <0.001 | 9.51(6.33,14.28) | <0.001 | 9.31(5.20,16.65) | <0.001 |
| Maternal outcomes | 4.63(3.64,5.87) | <0.001 | 7.80(5.85,10.41) | <0.001 | 14.11(9.59,20.78) | <0.001 | 18.24(10.47,31.78) | <0.001 |
| Referral cesarean delivery | 67.51(20.92,217.91) | <0.001 | 42.82(22.34,82.05) | <0.001 | 47.07(25.93,85.43) | <0.001 | 35.64(18.66,68.07) | <0.001 |
| Instrumental delivery | 5.55(3.54,8.72) | <0.001 | 8.81(5.42,14.32) | <0.001 | 12.71(7.29,22.18) | <0.001 | 9.83(4.66,20.76) | <0.001 |
| Postpartum hemorrhage | 2.78(1.80,4.30) | <0.001 | 3.85(2.35,6.32) | <0.001 | 7.10(4.10,12.27) | <0.001 | 8.28(4.11,16.68) | <0.001 |
| III and IV degree laceration | 2.41(1.21,4.83) | 0.01 | 4.09(1.87,8.96) | <0.001 | 5.45(2.13,13.94) | <0.001 | 9.67(3.45,27.08) | <0.001 |
| Length of stay ≥90th | 6.23(4.54,8.54) | <0.001 | 9.96(7.04,14.11) | <0.001 | 14.31(9.40,21.78) | <0.001 | 16.18(9.28,28.22) | <0.001 |
| Neonatal outcomes | 1.61(1.30,2.00) | <0.001 | 1.75(1.30,2.35) | <0.001 | 2.48(1.68,3.66) | <0.001 | 1.48(0.81,2.73) | 0.21 |
| NICU | 2.52(1.76,3.61) | <0.001 | 3.18(2.01,5.02) | <0.001 | 4.65(2.68,8.09) | <0.001 | 2.85(1.18,6.87) | 0.02 |
| Shoulder dystocia | 1.00(0.59,1.69) | 0.995 | 0.53(0.21,1.35) | 0.18 | 0.43(0.10,1.84) | 0.26 | 0(0,.) | 0.997 |
| Apgar ≤7(5 min) | 6.68(3.30,13.54) | <0.001 | 7.83(3.84,15.99) | <0.001 | 14.76(6.92,31.51) | <0.001 | 4.27(1.24,14.73) | 0.02 |
| Neonatal resuscitation | 1.48(1.15,1.90) | 0.002 | 1.68(1.20,2.36) | 0.003 | 2.40(1.56,3.69) | <0.001 | 1.40(0.70,2.81) | 0.34 |
| Assisted ventilation | 2.96(1.84,4.77) | <0.001 | 4.38(2.55,7.54) | <0.001 | 6.36(3.42,11.82) | <0.001 | 2.65(0.92,7.59) | 0.07 |

Multivariable logistic regression model was used to assess risks of cutoff value of the stage of labor for adverse delivery outcomes. Adjusted gestational age, maternal age, maternal height, maternal BMI, gravidity, parity, baby weight, baby height, epidural, anesthesia, induction, oxytocin.
